# Heterochromatic silencing in *Drosophila melanogaster*

**DOI:** 10.1186/1756-8935-6-S1-O26

**Published:** 2013-03-18

**Authors:** Sarah CR Elgin, Tingting Gu, Kiri Ulmschneider

**Affiliations:** 1 Washington University in St Louis, St Louis, MO 63130, USA

## 

Heterochromatin formation serves both to help organize and package large eukaryotic genomes, and to silence many of the transposable elements (TEs) that are an abundant component of these genomes. A critical question is how the cell decides which domains should be packaged in this form. Our prior studies on the heterochromatic fourth chromosome in *Drosophila melanogaster* have implicated the transposable element *1360* as a target for heterochromatin formation. *1360* remnants are concentrated in heterochromatic domains. Using a P element landing-pad construct to transpose TEs into the genome, we find that a euchromatic site close to a heterochromatic mass can be targeted for ectopic heterochromatin formation, as shown both by deposition of HP1a (ChIP-qPCR) and by the variegated silencing of the associated *hsp70-white* reporter gene. This outcome is achieved using either *1360* (a DNA transposon remnant) or *Invader4* (a retrotransposon remnant) as the target TE. The effect is dependent on piRNA sites within the TE. The piRNA system is most active in the developing oocyte and early embryo. We find that knock-down of *piwi* or *aubergine* in the female germ line results in over-expression of many, but not all, TEs, with associated loss of HP1a and H3K9me2 from these sites. Depletion of Piwi in the maternal germ line and early zygote leads to a suppression of position effect variegation (PEV) from *β-gal* reporters assayed in the adult, while knock-down of *piwi* in the somatic cells of the developing eye linage has no such effect on an *hsp70-white* reporter. In contrast, depletion of HP1a at any stage (maternal germ line, early embryo, somatic cell linage) will cause a suppression of PEV as assayed in the adult. We propose a model in which the assembly of heterochromatin at PEV reporters in the early zygote is dependent on the RNAi system as well as structural heterochromatin proteins, with failure producing an effect that persists in the mature animal. In contrast, maintenance of heterochromatin once set appears not to require RNAi (specifically Piwi), but does require the structural proteins, specifically HP1a. piRNA-directed heterochromatin formation appears to be a significant mechanism for down-regulating transcription from some (not all) TEs, and a significant mechanism for targeting the silencing of PEV reporters. Supported by NIH grant GM068388.

